# Ovarian Volume Correlates Strongly with the Number of Nongrowing Follicles in the Human Ovary

**DOI:** 10.1155/2012/305025

**Published:** 2012-02-12

**Authors:** Thomas W. Kelsey, W. Hamish B. Wallace

**Affiliations:** ^1^School of Computer Science, University of St. Andrews, St. Andrews, Scotland, Kelsey KY16 9SX, UK; ^2^Division of Reproductive and Developmental Sciences, University of Edinburgh, Edinburgh, Scotland, Wallace EH9 1LW, UK

## Abstract

A reliable indirect measure of ovarian reserve for the individual woman remains a challenge for reproductive specialists. Using descriptive statistics from a large-scale study of ovarian volumes, we have developed a normative model for healthy females for ages 25 through 85. For average values, this model has a strong and positive correlation (*r* = 0.89) with our recent model of nongrowing follicles (NGFs) in the human ovary for ages 25 through 51. When both models are log-adjusted, the correlation increases to *r* = 0.99, over the full range of ovarian volume. Furthermore we can deduce that an ovary of 3 cm^3^ volume (or less) contains approximately 1000 NGF (or fewer). These strong correlations indicate that ovarian volume is a useful factor in the indirect estimation of human ovarian reserve for the individual woman.

## 1. Introduction

The human ovary contains a fixed pool of nongrowing follicles (NGFs) maximal at 18–22 weeks gestation that declines towards menopause when fewer than one thousand NGFs are present [1]. The age at menopause in western populations is 50-51 years on average [2, 3]. Recent socioeconomic changes have resulted in an increasing number of women delaying childbirth until later in life, when their fertility is significantly compromised compared to younger women. This has created significant pressure on fertility services and an increased demand for assisted conception treatments (ACTs).

The assessment of ovarian reserve for the individual woman remains problematic. Direct estimation of the number of NGFs remaining in an individual ovary is currently impossible *in vivo*. A number of physical and humoral factors have been investigated in isolation and in combination. The measurement of follicular stimulating hormone (FSH) in the early follicular phase of the menstrual cycle is an accurate indicator of ovarian function but is not a good predictor of time remaining to menopause [4]. More recently, anti-Mullerian hormone (AMH) has shown promise as a measure of ovarian reserve [5, 6], and transvaginal ultrasound estimation of antral follicle counts (AFCs) is both a useful indicator of ovarian function and reserve [7]. The role of ovarian volume in the assessment of ovarian reserve remains uncertain, with some studies suggesting that a reduced volume is a good predictor of poor outcome for assisted conception [8, 9].

Ovarian volumes increase exponentially from birth to pubertal ages and are believed to be at a maximum shortly after puberty [10, 11]. Many studies have been published using ovarian volumes taken from women either attending infertility clinics or having polycystic ovarian syndrome; Lass and Brinsden have published a detailed survey of these [12]. There have been few studies on the ovarian volumes of groups of women who approximate the healthy population. Most of these were small-scale studies, with exemplars taking measurements from 38 [13] and 377 [14] subjects. One study dominates in terms of size and scope. Pavlik et al. recorded 58,673 volumes from 13,963 subjects taking part in an ovarian cancer-screening project [15]. Each ovary was measured in three dimensions via transvaginal ultrasound. Volumes were calculated using the formula for a prolate ellipsoid: L × H × W × 0.523. We consider this study to be the most comprehensive and therefore use it as our sole reference for ovarian volumes.

The aim of this paper is to correlate ovarian volumes as measured by transvaginal ultrasound with our recent description of the decline of the NGF population in the human ovary. Lass and Brinsden have shown that women with small ovaries (less than 3 mL) had a more than 50% risk of abandonment of IVF cycle before NGF retrieval, and that those who did not abandon required more aggressive stimulation than normal [12]. A close, positive correlation between volumes and NGF counts would provide further evidence that women with small ovaries (irrespective of age) are less likely to respond well to ACT. 

## 2. Materials and Methods

Summary statistics were extracted from the Pavlik et al. study [15]: for each year of age from 25 through 85 we obtained the mean ovarian volume, the upper standard deviation, and the number of observations. The total number of observations was 58,255. We derived the log-normal mean and standard deviation for each year of age using standard equations. Parametric bootstrapping is a standard statistical technique for simulating datapoints from a known distribution [16]. The *R* statistical package (The *R* Foundation for Statistical Computing, Vienna, Austria) has a parametric bootstrapping function that returns a fixed number of random deviates from given log-normal means and standard deviations. We used this function to create 10 datasets each having the same descriptive statistics as the Pavlik et al. study (so that each dataset reproduces their published results).

For ages 25–85, inspection of the Pavlik et al. plots shows that ovarian volumes appear to progress from high values declining to a minimum with increasing age. We therefore fitted seven sigmoidal (or “S-shaped”) models to the datasets using TableCurve2D (Systat Software Inc., Chicago, IL, USA) and ranked the returned models by the *r* ^2^ coefficient of determination. We also fitted 3,164 arbitrary and biologically nonplausible models to the same datasets in order to determine the maximum *r* ^2^ obtainable for that data.

We produced a normative model of ovarian volumes from the highest-ranked sigmoidal models and calculated the correlation coefficient, *r*, for mean ovarian volume against mean NGF population as given by the Wallace-Kelsey model [1] for ages 25 through 51 (since the highest age used in the derivation of the NGF model was 51 years, and the lowest age in the Pavlik et al. study was 25 years). For this correlation, neither quantity was log-adjusted. Variability increases with both ovarian volume and NGF population; hence both quantities are log-normally distributed. Therefore, in order to test correlation between predictive intervals as well as mean values, we log-adjusted both models and calculated correlation coefficients for mean values and upper and lower 95% prediction intervals (mean plus or minus 1.94 standard deviations) for the models.

## 3. Results

The curve fitting results are shown in Table 1. For each dataset a similar sigmoidal model gave the best fit. The *r* ^2^ coefficients of determination were typically 1% below the highest *r* ^2^ obtained for any model, indicating that our biologically plausible choice of sigmoidal model does not lead to significant under-fitting for these data.

**Figure 1:**
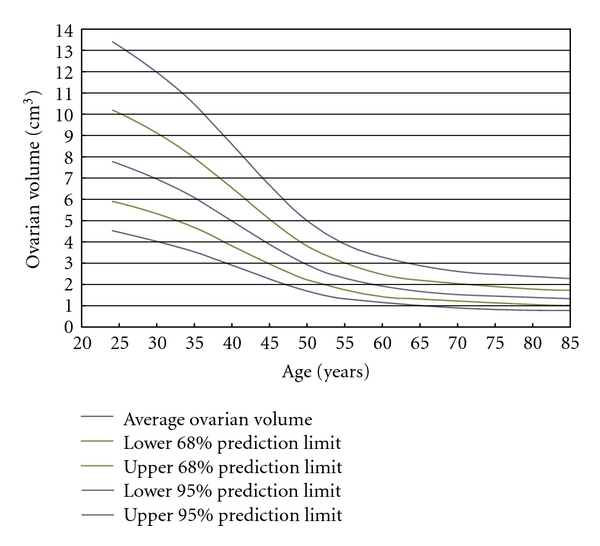
A normative model for the decline in human ovarian volumes from age 25. The centre line is the mean expected value for a given age. 68% of human ovarian volumes calculated at a known age are expected to fall within the lines at 1SD either side of the mean; 95% are expected to fall within the outer lines at 2SD from the mean.

Since there were no large-scale variations for any of the datasets, we report a three-parameter cumulative Lorentzian normative model of ovarian volume given by

(1) $${\log\;}_{10}\;\left(\text{ovarian}\;\text{volume}\right)=\frac{a}{\pi }\left(\arctan\left(\frac{\text{age}-b}{c}\right)+\frac{\pi }{2}\right)$$

with height parameter *a* = 1.08 (95% CI 1.02 through 1.13), centre parameter *b* = 46.9 (95% CI 45.4 through 48.3), and width parameter *c* = −13.7 (95% CI −14.6 through −12.9). This model can be interpreted as rapid decline in human ovarian volume from about age 33 to about age 61, with the rate of decline slowing after about age 47. A log-unadjusted version of the model is given in Figure 1, together with intervals in which the ovarian volumes of 68% and 95% of the population are expected to fall (mean plus or minus one or two standard deviations, resp.). 

**Figure 2:**
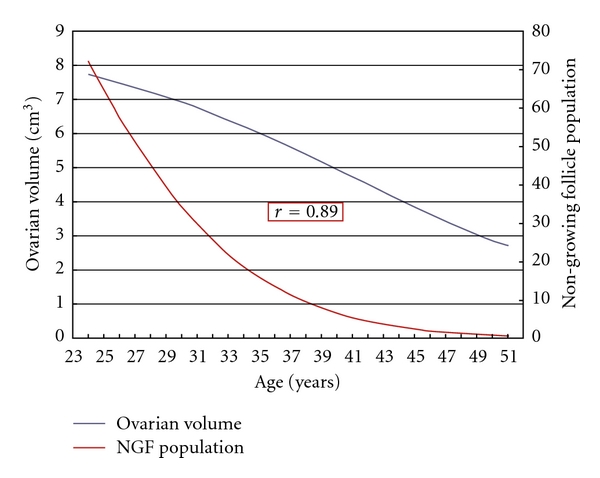
A strong and positive correlation, *r* = 0.89, between log-unadjusted mean ovarian volumes and mean NGF populations given by the Wallace-Kelsey model [1].

**Figure 3:**
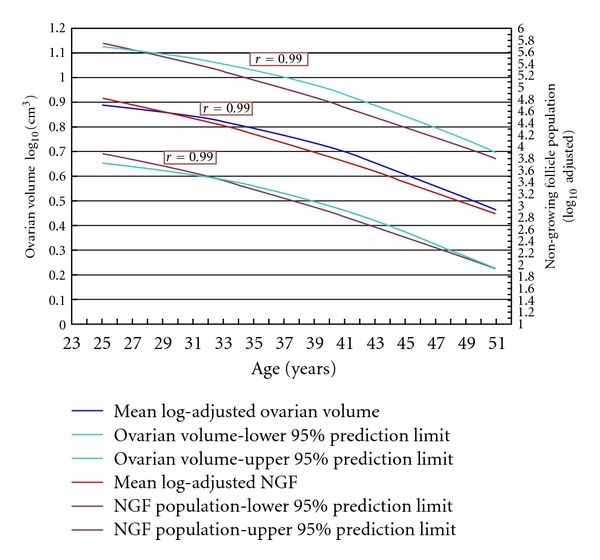
Extremely strong and positive correlations, *r* = 0.99 for each pair of lines, between log-adjusted ovarian volumes and the Wallace-Kelsey model of NGF population [1]. The inner lines are mean values; the outer lines are 95% prediction limits for the respective values. An NGF population of 1,000 (i.e., 10^3^) corresponds to an ovarian volume of 3.01 cm^3^ (i.e., 10^0.48^).

The correlation of mean ovarian volumes against mean NGF population, for ages 25 through 51, is given in Figure 2. We report a strong and positive correlation, *r* = 0.89, for this age range. For log-adjusted values of both ovarian volumes and NGF populations, we report extremely strong and positive correlations, *r* = 0.99, both for mean values and for decile, quartile, and percentual prediction limits.  Figure 3 illustrates this for mean values and 95% prediction limits. Using this correlation over all ranges of variation from average, we can infer that a population of 1000 NGFs (i.e., 10^3^) at any age represents approximately 3 cm^3^ volume (i.e., 10^0.48^).

## 4. Discussion

We have shown that there is a strong and positive correlation between ovarian volume and NGF population in the human ovary. We can therefore hypothesise that small ovaries have reduced numbers of NGFs and furthermore can calculate the number of NGFs in an ovary of known volume. We have also shown that an NGF population of one thousand corresponds to an ovarian volume of 3.01 cm^3^.

 Our results provide a simple method for assessment of remaining NGF pool for an individual. First obtain an accurate measurement of ovarian volume (by taking the average of two or more transvaginal ultrasound measurements, as set out in [17]). Use the base-10 logarithm of this value and the age of the individual to enter a datapoint on Figure 3. Read the value for this datapoint from the secondary *y*-axis, and raise 10 to a power of this value. The resulting number is an estimate of the number of NGFs remaining in that ovary.

Previous studies have shown that for women over 34 years of age, ovarian volume correlates strongly with follicular density in cortical tissue [9], and that large ovarian volumes are associated with good assisted reproductive technology outcomes whereas small ovarian volumes are associated with poor outcomes [8]. Our results agree with both sets of findings and also provide the quantitative information needed to say what “large” and “small” ovaries mean for the healthy population. Large (resp., small) ovaries at a known age have volumes greater (resp., less) than 1 SD from the average; very large and small ovaries are more than 2 SD away from average (Figure 1). This study extends and improves a similar study by Wallace and Kelsey [18] that reported a strong correlation between an earlier model of NGF decline with age and the mean ovarian volumes given by Pavlik et al. In this paper we have used an improved NGF model and have correlated not only average values but also all variations in both ovarian volume and NGF population.

Since data of this size and distribution provide similar goodness-of-fit results for the models tested (Table 1), and since each of our datasets provides the same results as the original paper, we have made the important assumption that the ten generated datasets accurately represent the original dataset [15].

We acknowledge a number of specific study limitations, the most important of which is that no causal link has been shown that explains the high correlation coefficients obtained in this study: it may be the case that a large ovary contains a large number of NGFs (in general) as our results suggest, but there is no direct evidence for this. Bowen et al. [19] have shown that reduced ovarian volumes predict reduced ovarian reserve (in terms of increased FSH) for infertile women, but we know that there are no studies that translate this finding to the fertile (normal) population.

A further limitation is that our ovarian volume model has not been validated: we have not tested how well or poorly the model generalises to unseen data. Indirect evidence for the validity of our model is given by Holm et al. [20]. This study produced a normative model of ovarian volumes for ages ranging from birth to 26 years, with a predicted average volume at age 24 of 7.8 mL. This entirely separate calculation agrees exactly with ours: we also predict an average volume of 7.8 mL at 24 years of age. Our model also agrees in qualitative terms (i.e., curvilinear decline at ages of menopause, followed by linear decline at older ages) with the normative model for postmenopausal ovarian volumes produced by Tepper et al. [21]. Our model reports smaller means and ranges than this study, due either to systematic differences in volume calculation between the two research groups or to the much smaller sample size (*n* = 311) for Tepper et al.

## 5. Conclusions

We have presented a normative model for human ovarian reserve for ages 25 through 85 and demonstrated that about two thirds of the variation in ovarian volumes for this age range is due to age alone. If our model were to be externally validated, and if it were shown that (in general) larger ovaries contain more NGFs than smaller ones (possibly by inference from a mammalian model), then the strong and positive correlations that we have reported indicate that the remaining follicle pool for individuals can be accurately assessed using ovarian volumes as a surrogate measure. Moreover, our model can be correlated against other models of indirect measures of ovarian reserve, such as the normative model for serum AMH in the healthy population [22, 23]. We speculate that a multivariate model can be derived, involving both endocrine factors and physiological factors (such as ovarian volume and antral follicle count), that will allow the accurate estimation of remaining fertile lifespan for individual women. This would have major implications for the planning of assisted conception cycles and for the preservation of fertility for survivors of cancer earlier in life.

## Figures and Tables

**Table 1:** Curve-fitting results for 10 constructed datasets.

Dataset	Highest-ranked sigmoidal model				Highest-ranked model	
	Height	Centre	Width	*r* ^2^	Parameters	*r* ^2^
1	1.07	47.1	−13.7	0.667	13	0.678
2	1.07	47.0	−13.6	0.671	12	0.681
3	1.08	46.9	−13.7	0.669	12	0.679
4	1.07	47.0	−13.6	0.669	13	0.680
5	1.07	47.1	−13.6	0.672	13	0.682
6	1.08	46.9	−13.7	0.669	12	0.683
7	1.08	46.8	−13.6	0.669	12	0.680
8	1.07	47.0	−13.7	0.672	12	0.682
9	1.07	46.9	−13.7	0.668	13	0.678
10	1.07	47.0	−13.7	0.668	12	0.678
